# 6-Methyl-2,3,4,9-tetra­hydro-1*H*-carbazole-1-thione

**DOI:** 10.1107/S1600536811019246

**Published:** 2011-06-11

**Authors:** R. Archana, K. Prabakaran, K. J. Rajendra Prasad, A. Thiruvalluvar, R. J. Butcher

**Affiliations:** a PG Research Department of Physics, Rajah Serfoji Government College (Autonomous), Thanjavur 613 005, Tamilnadu, India; b Department of Chemistry, Bharathiar University, Coimbatore 641 046, Tamilnadu, India; c Department of Chemistry, Howard University, 525 College Street NW, Washington, DC 20059, USA

## Abstract

In the title mol­ecule, C_13_H_13_NS, the dihedral angle between the benzene ring and the fused pyrrole ring is 0.71 (8)° and the cyclo­hexene ring is in an envelope form. The (CH_2_)_3_ atoms of the cyclo­hexene ring are disordered over two positions; the site-occupancy factor for the major component refined to 0.862 (4). In the crystal, inter­molecular N—H⋯S hydrogen bonds lead to the formation of centrosymmetric aggregates *via* an *R*_2_^2^(10) ring.

## Related literature

For the synthesis of fused carbazole nuclei, see: Pelly *et al.* (2005 ▶). For heterocycle-annulated tetra-, penta- and hexa­cyclic carbazole derivatives, see: Chattopadhyay *et al.* (2006 ▶). For the preparation of 1-oxo compounds *via* their corresponding hydrazones, see: Rajendra Prasad & Vijayalakshmi (1994 ▶). For related structures, see: Archana *et al.* (2010 ▶); Thomas Gunaseelan *et al.* (2009 ▶). For hydrogen-bond motifs, see: Bernstein *et al.* (1995 ▶).
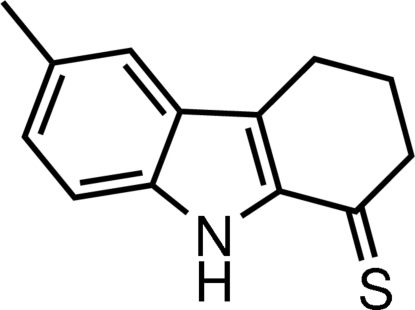


## Experimental

### 

#### Crystal data

C_13_H_13_NS
*M* *_r_* = 215.31
Triclinic, 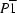

*a* = 7.0846 (4) Å
*b* = 9.5287 (7) Å
*c* = 9.6384 (6) Å
α = 115.009 (7)°
β = 104.901 (6)°
γ = 98.074 (6)°
*V* = 546.28 (8) Å^3^
*Z* = 2
Cu *K*α radiation
μ = 2.31 mm^−1^
*T* = 295 K
0.46 × 0.28 × 0.21 mm

#### Data collection

Oxford Diffraction Xcalibur Ruby Gemini diffractometer
Absorption correction: multi-scan (*CrysAlis PRO*; Oxford Diffraction, 2010 ▶) *T*_min_ = 0.609, *T*_max_ = 1.000
3471 measured reflections
2102 independent reflections
1924 reflections with *I* > 2σ(*I*)
*R*_int_ = 0.022

#### Refinement

*R*[*F* ^2^ > 2σ(*F* ^2^)] = 0.045
*wR*(*F* ^2^) = 0.133
*S* = 1.06
2102 reflections
145 parameters
3 restraints
H atoms treated by a mixture of independent and constrained refinement
Δρ_max_ = 0.33 e Å^−3^
Δρ_min_ = −0.22 e Å^−3^

### 

Data collection: *CrysAlis PRO* (Oxford Diffraction, 2010 ▶); cell refinement: *CrysAlis PRO*; data reduction: *CrysAlis PRO*; program(s) used to solve structure: *SHELXS97* (Sheldrick, 2008 ▶); program(s) used to refine structure: *SHELXL97* (Sheldrick, 2008 ▶); molecular graphics: *ORTEP-3* (Farrugia, 1997 ▶) and *PLATON* (Spek, 2009 ▶); software used to prepare material for publication: *PLATON*.

## Supplementary Material

Crystal structure: contains datablock(s) global, I. DOI: 10.1107/S1600536811019246/tk2746sup1.cif

Structure factors: contains datablock(s) I. DOI: 10.1107/S1600536811019246/tk2746Isup2.hkl

Supplementary material file. DOI: 10.1107/S1600536811019246/tk2746Isup3.cml

Additional supplementary materials:  crystallographic information; 3D view; checkCIF report

## Figures and Tables

**Table 1:** Hydrogen-bond geometry (Å, °)

*D*—H⋯*A*	*D*—H	H⋯*A*	*D*⋯*A*	*D*—H⋯*A*
N9—H9⋯S1^i^	0.86 (2)	2.77 (3)	3.4955 (15)	143 (2)

Symmetry code: (i) 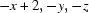.

**Fig. 1.**
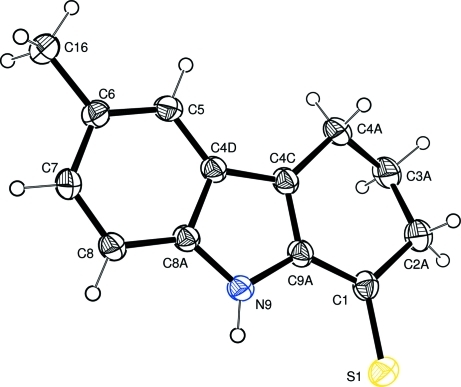
The molecular structure of the title compound, showing the atom-numbering scheme and displacement ellipsoids drawn at the 30% probability level. H atoms are shown as small spheres of arbitrary radius.

**Fig. 2.**
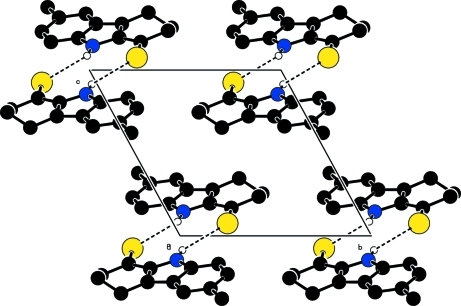
Unit cell contents for (I), viewed down the a axis, showing the formation of a R22(10) ring.

## supplementary crystallographic information

### Comment

The development of methods for the synthesis of fused carbazole nuclei is becoming increasingly important as a result of the number of natural and synthetic carbazoles that display biological activity (Pelly *et al.*, 2005). Heterocycle-annulated tetra-, penta- and hexa-cyclic carbazole derivatives have been developed using successive applications of three atom economic processes, *viz*., Claisen rearrangement, olefin metathesis and Diels-Alder reactions (Chattopadhyay *et al.*, 2006). The preparation of 1-oxo compounds *via* their corresponding hydrazones has been reported (Rajendra Prasad & Vijayalakshmi, 1994). Archana *et al.* (2010) and Thomas Gunaseelan *et al.* (2009) have reported the crystal structures of substituted carbazole derivatives, in which the carbazole units are not planar.

In the title molecule, Fig. 1, the dihedral angle between the benzene ring and the fused pyrrole ring is 0.71 (8) °. The cyclohexene ring is in envelope form. Three C atoms (C2A, C3A, C4A) of the cyclohexene ring, with their attached H atoms are disordered over two positions; the site-occupancy factors are *ca* 0.86 and 0.14. Intermolecular N—H···S hydrogen bonds form a *R*^2^_2_(10) (Bernstein *et al.*, 1995) ring in the crystal structure (Table 1 & Fig. 2).

### Experimental

A mixture of 6-methyl-2,3,4,9-tetrahydro-1*H*-carbazol-1-one (0.199 g, 0.001 mol) and Lawesson's reagent (0.404 g, 0.001 mol) was refluxed in pyridine on an oil bath pre-heated to 383 K for 6 h. The contents were poured onto cold water and neutralized using 1:1 HCl, filtered and dried. The product was recrystallized from ethanol. The yield was 0.154 g (72%).

### Refinement

Atoms C2A, C3A, C4A of the cyclohexene ring, with attached hydrogen atoms are disordered over two positions; the site occupancy factors refined to 0.862 (4) and 0.138 (4). The N9-H atom was located in a difference Fourier map and refined freely. Other H atoms were positioned geometrically and allowed to ride on their parent atoms, with C—H = 0.93–0.97 Å and *U*_iso_(H) = *xU*_eq_(parent atom), where *x* = 1.5 for methyl and 1.2 for all other carbon-bound H atoms. A damping factor (damp 200 15 in the final refinement cycles) was applied to avoid large and erratic displacements of the hydrogen atoms of the less occupied C atoms.

### Crystal data

**Table:**

C_13_H_13_NS	*Z* = 2
*M**_r_* = 215.31	*F*(000) = 228
Triclinic, *P*1	*D*_x_ = 1.309 Mg m^−^^3^
Hall symbol: -P 1	Melting point: 356 K
*a* = 7.0846 (4) Å	Cu *K*α radiation, λ = 1.54184 Å
*b* = 9.5287 (7) Å	Cell parameters from 2595 reflections
*c* = 9.6384 (6) Å	θ = 5.3–72.6°
α = 115.009 (7)°	µ = 2.31 mm^−^^1^
β = 104.901 (6)°	*T* = 295 K
γ = 98.074 (6)°	Chunk, orange
*V* = 546.28 (8) Å^3^	0.46 × 0.28 × 0.21 mm

### Data collection

**Table:**

Oxford Diffraction Xcalibur Ruby Gemini diffractometer	2102 independent reflections
Radiation source: Enhance (Cu) X-ray Source	1924 reflections with *I* > 2σ(*I*)
graphite	*R*_int_ = 0.022
Detector resolution: 10.5081 pixels mm^-1^	θ_max_ = 72.8°, θ_min_ = 5.3°
ω scans	*h* = −7→8
Absorption correction: multi-scan (*CrysAlis PRO*; Oxford Diffraction, 2010)	*k* = −11→11
*T*_min_ = 0.609, *T*_max_ = 1.000	*l* = −9→11
3471 measured reflections	

### Refinement

**Table:**

Refinement on *F*^2^	Primary atom site location: structure-invariant direct methods
Least-squares matrix: full	Secondary atom site location: difference Fourier map
*R*[*F*^2^ > 2σ(*F*^2^)] = 0.045	Hydrogen site location: inferred from neighbouring sites
*wR*(*F*^2^) = 0.133	H atoms treated by a mixture of independent and constrained refinement
*S* = 1.06	*w* = 1/[σ^2^(*F*_o_^2^) + (0.0834*P*)^2^ + 0.089*P*] where *P* = (*F*_o_^2^ + 2*F*_c_^2^)/3
2102 reflections	(Δ/σ)_max_ = 0.001
145 parameters	Δρ_max_ = 0.33 e Å^−^^3^
3 restraints	Δρ_min_ = −0.22 e Å^−^^3^

### Special details

**Table:**

Geometry. Bond distances, angles *etc*. have been calculated using the rounded fractional coordinates. All su's are estimated from the variances of the (full) variance-covariance matrix. The cell e.s.d.'s are taken into account in the estimation of distances, angles and torsion angles
Refinement. Refinement of *F*^2^ against ALL reflections. The weighted *R*-factor *wR* and goodness of fit *S* are based on *F*^2^, conventional *R*-factors *R* are based on *F*, with *F* set to zero for negative *F*^2^. The threshold expression of *F*^2^ > 2σ(*F*^2^) is used only for calculating *R*-factors(gt) *etc*. and is not relevant to the choice of reflections for refinement. *R*-factors based on *F*^2^ are statistically about twice as large as those based on *F*, and *R*- factors based on ALL data will be even larger.

### Fractional atomic coordinates and isotropic or equivalent isotropic displacement parameters (Å^2^)

**Table:**

	*x*	*y*	*z*	*U*_iso_*/*U*_eq_	Occ. (<1)
S1	1.12016 (7)	0.28362 (5)	0.07548 (6)	0.0567 (1)	
N9	0.79545 (19)	0.08614 (14)	0.14611 (15)	0.0412 (3)	
C1	0.9615 (3)	0.34787 (18)	0.16910 (19)	0.0438 (4)	
C2A	0.9571 (3)	0.5227 (2)	0.2371 (3)	0.0611 (6)	0.862 (4)
C3A	0.7579 (4)	0.5495 (2)	0.2548 (3)	0.0590 (7)	0.862 (4)
C4A	0.6807 (3)	0.47256 (19)	0.3468 (2)	0.0531 (5)	0.862 (4)
C4C	0.6904 (3)	0.30147 (17)	0.27848 (18)	0.0421 (4)	
C4D	0.5783 (2)	0.16637 (17)	0.27860 (17)	0.0396 (4)	
C5	0.4231 (3)	0.14228 (19)	0.33927 (19)	0.0443 (4)	
C6	0.3399 (2)	−0.00860 (19)	0.31681 (18)	0.0430 (4)	
C7	0.4130 (2)	−0.13824 (18)	0.23121 (19)	0.0444 (4)	
C8	0.5640 (2)	−0.12039 (18)	0.16959 (19)	0.0426 (4)	
C8A	0.6473 (2)	0.03337 (17)	0.19299 (17)	0.0382 (4)	
C9A	0.8222 (2)	0.24863 (17)	0.19625 (18)	0.0406 (4)	
C16	0.1720 (3)	−0.0386 (2)	0.3790 (2)	0.0530 (5)	
C4B	0.6807 (3)	0.47256 (19)	0.3468 (2)	0.0531 (5)	0.138 (4)
C3B	0.855 (2)	0.5809 (14)	0.3534 (18)	0.0590 (7)	0.138 (4)
C2B	0.9571 (3)	0.5227 (2)	0.2371 (3)	0.0611 (6)	0.138 (4)
H3A	0.65603	0.50556	0.14672	0.0708*	0.862 (4)
H2B	0.98737	0.56293	0.16579	0.0733*	0.862 (4)
H4B	0.54131	0.47484	0.33584	0.0637*	0.862 (4)
H3B	0.77491	0.66471	0.31193	0.0708*	0.862 (4)
H4A	0.76376	0.53281	0.46218	0.0637*	0.862 (4)
H8	0.60931	−0.20752	0.11426	0.0512*	
H9	0.856 (3)	0.029 (3)	0.086 (2)	0.050 (5)*	
H16A	0.14149	0.06091	0.43500	0.0795*	
H16B	0.21516	−0.08010	0.45319	0.0795*	
H16C	0.05241	−0.11568	0.28852	0.0795*	
H5	0.37662	0.22852	0.39473	0.0531*	
H7	0.35612	−0.24001	0.21612	0.0532*	
H2A	1.06434	0.58601	0.34358	0.0733*	0.862 (4)
H2C	1.09697	0.59042	0.28888	0.0733*	0.138 (4)
H2D	0.89345	0.53906	0.14537	0.0733*	0.138 (4)
H3C	0.80970	0.66932	0.34463	0.0708*	0.138 (4)
H3D	0.95675	0.62673	0.46174	0.0708*	0.138 (4)
H4C	0.55597	0.47686	0.27954	0.0637*	0.138 (4)
H4D	0.67683	0.50937	0.45626	0.0637*	0.138 (4)

### Atomic displacement parameters (Å^2^)

**Table:**

	*U*^11^	*U*^22^	*U*^33^	*U*^12^	*U*^13^	*U*^23^
S1	0.0577 (2)	0.0544 (2)	0.0691 (3)	0.0166 (2)	0.0349 (2)	0.0319 (2)
N9	0.0486 (6)	0.0350 (6)	0.0475 (6)	0.0156 (5)	0.0246 (5)	0.0209 (5)
C1	0.0476 (8)	0.0392 (7)	0.0458 (7)	0.0084 (6)	0.0155 (6)	0.0232 (6)
C2A	0.0759 (11)	0.0386 (8)	0.0776 (11)	0.0143 (8)	0.0367 (9)	0.0307 (7)
C3A	0.0760 (14)	0.0372 (8)	0.0728 (13)	0.0214 (9)	0.0301 (11)	0.0304 (9)
C4A	0.0659 (10)	0.0342 (7)	0.0616 (9)	0.0190 (7)	0.0300 (8)	0.0194 (6)
C4C	0.0506 (8)	0.0340 (7)	0.0432 (7)	0.0125 (6)	0.0184 (6)	0.0187 (5)
C4D	0.0474 (7)	0.0338 (6)	0.0402 (6)	0.0132 (5)	0.0179 (6)	0.0180 (5)
C5	0.0517 (8)	0.0407 (7)	0.0453 (7)	0.0181 (6)	0.0239 (6)	0.0194 (6)
C6	0.0440 (7)	0.0451 (7)	0.0416 (7)	0.0113 (6)	0.0178 (6)	0.0211 (6)
C7	0.0493 (8)	0.0363 (7)	0.0497 (7)	0.0093 (6)	0.0188 (6)	0.0226 (6)
C8	0.0504 (8)	0.0342 (6)	0.0468 (7)	0.0147 (6)	0.0206 (6)	0.0197 (5)
C8A	0.0439 (7)	0.0347 (6)	0.0389 (6)	0.0132 (5)	0.0167 (5)	0.0183 (5)
C9A	0.0481 (8)	0.0339 (6)	0.0426 (7)	0.0119 (6)	0.0174 (6)	0.0200 (5)
C16	0.0521 (9)	0.0552 (9)	0.0545 (8)	0.0109 (7)	0.0256 (7)	0.0262 (7)
C4B	0.0659 (10)	0.0342 (7)	0.0616 (9)	0.0190 (7)	0.0300 (8)	0.0194 (6)
C3B	0.0760 (14)	0.0372 (8)	0.0728 (13)	0.0214 (9)	0.0301 (11)	0.0304 (9)
C2B	0.0759 (11)	0.0386 (8)	0.0776 (11)	0.0143 (8)	0.0367 (9)	0.0307 (7)

### Geometric parameters (Å, °)

**Table:**

S1—C1	1.643 (2)	C7—C8	1.374 (2)
N9—C8A	1.359 (2)	C8—C8A	1.398 (3)
N9—C9A	1.380 (2)	C2A—H2A	0.9700
N9—H9	0.86 (2)	C2A—H2B	0.9700
C1—C9A	1.420 (3)	C2B—H2C	0.9700
C1—C2B	1.519 (3)	C2B—H2D	0.9700
C1—C2A	1.519 (3)	C3A—H3A	0.9700
C2A—C3A	1.508 (4)	C3A—H3B	0.9700
C2B—C3B	1.446 (15)	C3B—H3C	0.9700
C3A—C4A	1.520 (3)	C3B—H3D	0.9700
C3B—C4B	1.463 (15)	C4A—H4A	0.9700
C4A—C4C	1.498 (3)	C4A—H4B	0.9700
C4B—C4C	1.498 (3)	C4B—H4D	0.9700
C4C—C4D	1.415 (3)	C4B—H4C	0.9700
C4C—C9A	1.389 (3)	C5—H5	0.9300
C4D—C5	1.406 (3)	C7—H7	0.9300
C4D—C8A	1.422 (2)	C8—H8	0.9300
C5—C6	1.376 (3)	C16—H16C	0.9600
C6—C16	1.507 (3)	C16—H16A	0.9600
C6—C7	1.419 (2)	C16—H16B	0.9600
			
C8A—N9—C9A	108.73 (13)	H2A—C2A—H2B	108.00
C8A—N9—H9	127.5 (19)	C1—C2B—H2C	108.00
C9A—N9—H9	123.4 (19)	C1—C2B—H2D	108.00
S1—C1—C2B	121.48 (16)	C3B—C2B—H2C	108.00
C2A—C1—C9A	114.66 (17)	C3B—C2B—H2D	108.00
C2B—C1—C9A	114.66 (17)	H2C—C2B—H2D	107.00
S1—C1—C2A	121.48 (16)	C2A—C3A—H3B	109.00
S1—C1—C9A	123.85 (14)	C4A—C3A—H3A	109.00
C1—C2A—C3A	114.80 (19)	C2A—C3A—H3A	109.00
C1—C2B—C3B	118.3 (6)	H3A—C3A—H3B	108.00
C2A—C3A—C4A	113.5 (2)	C4A—C3A—H3B	109.00
C2B—C3B—C4B	121.0 (10)	C2B—C3B—H3D	107.00
C3A—C4A—C4C	109.36 (17)	C2B—C3B—H3C	107.00
C3B—C4B—C4C	112.2 (6)	C4B—C3B—H3D	107.00
C4B—C4C—C9A	122.29 (17)	H3C—C3B—H3D	107.00
C4B—C4C—C4D	130.69 (18)	C4B—C3B—H3C	107.00
C4A—C4C—C4D	130.69 (18)	C3A—C4A—H4A	110.00
C4A—C4C—C9A	122.29 (17)	C3A—C4A—H4B	110.00
C4D—C4C—C9A	107.01 (15)	H4A—C4A—H4B	108.00
C4C—C4D—C5	134.04 (17)	C4C—C4A—H4A	110.00
C4C—C4D—C8A	106.52 (14)	C4C—C4A—H4B	110.00
C5—C4D—C8A	119.43 (16)	C4C—C4B—H4D	109.00
C4D—C5—C6	120.16 (17)	H4C—C4B—H4D	108.00
C7—C6—C16	119.78 (17)	C3B—C4B—H4C	109.00
C5—C6—C16	121.41 (16)	C3B—C4B—H4D	109.00
C5—C6—C7	118.81 (16)	C4C—C4B—H4C	109.00
C6—C7—C8	123.03 (17)	C4D—C5—H5	120.00
C7—C8—C8A	117.67 (15)	C6—C5—H5	120.00
N9—C8A—C4D	108.52 (15)	C6—C7—H7	119.00
N9—C8A—C8	130.59 (15)	C8—C7—H7	118.00
C4D—C8A—C8	120.89 (14)	C7—C8—H8	121.00
N9—C9A—C4C	109.22 (15)	C8A—C8—H8	121.00
C1—C9A—C4C	124.73 (17)	C6—C16—H16A	109.00
N9—C9A—C1	126.04 (15)	C6—C16—H16B	109.00
C1—C2A—H2A	109.00	C6—C16—H16C	109.00
C1—C2A—H2B	109.00	H16A—C16—H16B	109.00
C3A—C2A—H2A	109.00	H16A—C16—H16C	109.00
C3A—C2A—H2B	109.00	H16B—C16—H16C	109.00
			
C9A—N9—C8A—C4D	−1.01 (16)	C4A—C4C—C9A—N9	−179.30 (14)
C9A—N9—C8A—C8	179.17 (15)	C4A—C4C—C9A—C1	0.7 (3)
C8A—N9—C9A—C1	−179.28 (15)	C4D—C4C—C9A—N9	−0.11 (17)
C8A—N9—C9A—C4C	0.70 (17)	C4D—C4C—C9A—C1	179.87 (15)
S1—C1—C2A—C3A	155.86 (17)	C4C—C4D—C5—C6	178.74 (17)
C9A—C1—C2A—C3A	−25.3 (3)	C8A—C4D—C5—C6	0.4 (2)
S1—C1—C9A—N9	−1.7 (2)	C4C—C4D—C8A—N9	0.93 (17)
S1—C1—C9A—C4C	178.38 (13)	C4C—C4D—C8A—C8	−179.22 (14)
C2A—C1—C9A—N9	179.56 (16)	C5—C4D—C8A—N9	179.65 (14)
C2A—C1—C9A—C4C	−0.4 (2)	C5—C4D—C8A—C8	−0.5 (2)
C1—C2A—C3A—C4A	51.1 (3)	C4D—C5—C6—C7	−0.3 (2)
C2A—C3A—C4A—C4C	−48.2 (2)	C4D—C5—C6—C16	−179.42 (15)
C3A—C4A—C4C—C4D	−155.34 (19)	C5—C6—C7—C8	0.2 (2)
C3A—C4A—C4C—C9A	23.7 (2)	C16—C6—C7—C8	179.32 (15)
C4A—C4C—C4D—C5	0.2 (3)	C6—C7—C8—C8A	−0.2 (2)
C4A—C4C—C4D—C8A	178.61 (16)	C7—C8—C8A—N9	−179.81 (15)
C9A—C4C—C4D—C5	−178.95 (17)	C7—C8—C8A—C4D	0.4 (2)
C9A—C4C—C4D—C8A	−0.50 (17)		

### Hydrogen-bond geometry (Å, °)

**Table:**

*D*—H···*A*	*D*—H	H···*A*	*D*···*A*	*D*—H···*A*
N9—H9···S1^i^	0.86 (2)	2.77 (3)	3.4955 (15)	143 (2)

Symmetry codes: (i) −*x*+2, −*y*, −*z*.
